# Molecular Regulation of Thermogenic Mechanisms in Beige Adipocytes

**DOI:** 10.3390/ijms25126303

**Published:** 2024-06-07

**Authors:** Siqi Yang, Yingke Liu, Xiaoxu Wu, Rongru Zhu, Yuanlu Sun, Shuoya Zou, Dongjie Zhang, Xiuqin Yang

**Affiliations:** 1 College of Animal Science and Technology, Northeast Agricultural University, Harbin 150030, China; siqiyang2001@163.com (S.Y.); liuyingke1998@163.com (Y.L.); wxiaox2022@163.com (X.W.); zhurongruzi@163.com (R.Z.); sunyuanlu2023@126.com (Y.S.); m1614369056@126.com (S.Z.); 2 Institute of Animal Husbandry, Heilongjiang Academy of Agricultural Sciences, Harbin 150086, China

**Keywords:** beige adipose tissue, thermogenic mechanisms, molecular regulation of thermogenesis

## Abstract

Adipose tissue is conventionally recognized as a metabolic organ responsible for storing energy. However, a proportion of adipose tissue also functions as a thermogenic organ, contributing to the inhibition of weight gain and prevention of metabolic diseases. In recent years, there has been significant progress in the study of thermogenic fats, particularly brown adipose tissue (BAT). Despite this progress, the mechanism underlying thermogenesis in beige adipose tissue remains highly controversial. It is widely acknowledged that beige adipose tissue has three additional thermogenic mechanisms in addition to the conventional UCP1-dependent thermogenesis: Ca^2+^ cycling thermogenesis, creatine substrate cycling thermogenesis, and triacylglycerol/fatty acid cycling thermogenesis. This paper delves into these three mechanisms and reviews the latest advancements in the molecular regulation of thermogenesis from the molecular genetic perspective. The objective of this review is to provide readers with a foundation of knowledge regarding the beige fats and a foundation for future research into the mechanisms of this process, which may lead to the development of new strategies for maintaining human health.

## 1. Introduction

Obesity is a significant health concern worldwide, and its increasing prevalence poses a severe threat to public health [1]. This is embodied in that obesity is associated with numerous chronic metabolic diseases, such as hypertension (HTN), type 2 diabetes mellitus (T2DM), hyperlipidemia (HLD), and non-alcoholic fatty liver disease (NAFLD) [2]. The root cause of obesity lies in a chronic imbalance between energy intake and expenditure, resulting in fat accumulation. Adipose tissue, as a metabolically active organ, serves as the primary energy reservoir [3]. Historically, it was recognized that mammals possess three distinct types of adipose tissue—white adipose tissue (WAT), brown adipose tissue (BAT), and beige adipose tissue [4]. Of these, the latter two types have a thermogenic function [5]. Thermogenic adipocytes (brown adipocytes and beige adipocytes) convert chemical energy derived from carbohydrates and fats into heat. These two adipocytes regulate body temperature in cold environments and facilitate the mobilization of excess fat away from storage [6]. Distinct from white adipocytes, the unique characteristics of thermogenic adipocytes include numerous small lipid droplets instead of a single large one and a high concentration of mitochondria, which are cristae-dense and express UCP1 (uncoupling protein 1). UCP1 catalyzes a proton leak across the inner membrane of the mitochondria, thereby uncoupling the oxidation of fuel from ATP synthesis. Functionally, BAT is involved in non-shivering heat production in the body and maintains core temperature in hibernating animals and newborn infants. Initially, it was thought to be absent in human adults. However, subsequent studies employing ^18^F-fluorodeoxyglucose positron emission tomography combined with computed tomography (^18^F-FDG-PET/CT) and biopsy demonstrated the presence of UCP1-expressing BAT in human adults [7].

It is notable that WAT also develops clusters of thermogenic UCP1-expressing adipocytes in response to various stimuli, including chronic cold acclimation, exercise, long-term treatment with PPARγ agonists or β3-adrenergic receptor (AR) agonist, cancer cachexia, and tissue injury. These adipocytes have been named beige, brite (brown in white), iBAT (induced BAT), recruitable BAT, and wBAT (white adipose BAT) [8]. Although brown adipocytes and beige adipocytes are similar in their ability to produce heat, there are still significant differences between them. First, when not stimulated, beige adipocytes behave like white adipocytes. In other words, it is dynamic in the presence or absence of irritation. Second, regarding heat production, in addition to classical UCP1-dependent thermogenesis, beige adipocytes can undergo non-shivering heat production via Ca^2+^ cycling thermogenesis, creatine substrate cycling thermogenesis, and triacylglycerol/fatty acid cycling thermogenesis. Third, the two types of thermogenic adipocytes differ in their developmental timing and lineage [9]. As such, as previously stated, it is inaccurate to identify adipose tissue as BAT solely based on the results of FDG-PET/CT and UCP1 expression. Based on this, it is advisable to conduct specific genetic testing for beige adipocytes.

While numerous studies have focused on BAT in the thermogenic adipose tissue, there is a scarcity of research on beige adipose tissue. However, studies investigating beige adipose tissue have promising potential, specifically regarding the following areas:

a. Beige adipocytes can be recruited throughout WAT by various mechanisms in adult humans and rodents, whereas BAT is typically observed only in early childhood [10].
b. Some animals, such as pigs, possess beige adipose tissue but lack BAT [11].
c. It is possible that beige adipocytes have more pathways for non-shivering thermogenesis beyond classical UCP1-dependent thermogenesis.
d. Animal bodies are constantly changing, at the same time, beige adipose tissue is also undergoing changes under different conditions.

Thus, beige adipocytes have emerged as a promising cellular target not only in non-shivering thermogenesis but also in metabolic homeostasis for glucose and free fatty acids to combat obesity and diabetes. Moreover, in the field of animal husbandry, there have been related studies on the cold tolerance of beige adipose tissue in pigs and the cold stress response in piglets.

## 2. Adipose Tissue Is Complicate and Heterogeneous

Adipose tissue is composed of mature adipocytes and a stromal vascular fraction (SVF) which includes adipose tissue-derived mesenchymal stem cells (ASCs), endothelial progenitor cells (EPCs), endothelial cells (ECs), macrophage, smooth muscle cells, pericyte, and so on [12,13,14], so it is highly malleable. The following is a further explanation for adipose tissue. Moreover, consequences of fat metabolism differ due to tissue-specific mechanisms [15].

### 2.1. Calcification of Adipose Tissue Based on the Position: Subcutaneous and Visceral Fat

Adipose tissue can be divided into subcutaneous fat (SAT) and visceral fat (VAT) based on distribution differences. Another study specifically shows SAT and VAT have different origins, with partial VAT originating from Wt1-positive precursor cells, while SAT originates from Wt1-negative precursor cells [16]. Regarding cytokines expression, both leptin and lipocalin are expressed at lower levels in VAT compared to SAT [17,18]. At the genetic level, the aggregation of VAT is related to the absence of storage genes in SAT, such as diacylgycerol acytransferase (DGAT2), sterol regulatory element binding protein-1c (SREBP-1c), cell death-inducing Dff45-like effecter A (CIDEA), and so on [19]. In some metabolic diseases, such as non-alcoholic fatty liver (NAFL) and steatohepatitis (NASH) in obese humans, significant reductions in mitochondrial respiration have been observed in VAT, whereas no such reductions are noted in SAT [20]. A recent study also reported that the circadian rhythm of these genes (SREBP-1c, FAS, LPL and CPT1A) that related to lipid synthesis and mobilization is different between SAT and VAT [21]. However, it is unclear whether there is a simple correlation or a causal relationship between distinct metabolic responses and regulation in VAT and SAT.

### 2.2. Classification of Adipose Tissue Based on Their Color and Morphology: White, Brown and Beige Adipose Tissue

Adipose tissue can be divided into white adipose tissue (WAT), brown adipose tissue (BAT), and beige adipose tissue according to their color and morphology. WAT is typically yellow in color, and in some mammals, such as mice, it appears white. WAT is a significant site for storing and mobilizing lipids. It stores energy from glucose and fatty acids in the form of triacylglycerol (TAG) and releases energy as free fatty acids (FFAs) [22].

BAT was first identified in marmots’ hibernating gland in 1551 [23,24]. However, the issue of BAT has not been effectively validated, because BAT is primarily present in newborns and rodents and is rarely found in adults. In 2009, the distribution of BAT in the supraclavicular, cervical, axillary, mediastinal, paraspinal and abdominal depots of the adult uterus has been confirmed by 18F-FDG-PET/CT [25,26]. BAT is rich in mitochondria and expresses uncoupling protein 1 (UCP1), which can dissipate energy in the form of heat [27]. Therefore, UCP1 serves as a significant marker for identifying BAT.

In 1992, beige adipose tissue was identified as a special type of adipose tissue in the WAT of rats [28]. Under warm conditions, beige adipocytes show similar morphology and function to white adipocytes. However, in response to cold stimuli or exposure to β-adrenoceptor agonist, beige adipocytes demonstrate a significant enhancement in their thermogenic capacity with *UCP1* expression approaching levels comparable to those of classic brown adipocytes [29]. Although both BAT and beige adipose tissue are capable of producing heat, there are differences in their origins, forms of existence, and mechanisms of action.

## 3. Origin of Thermogenic Adipocytes

**Figure 1:**
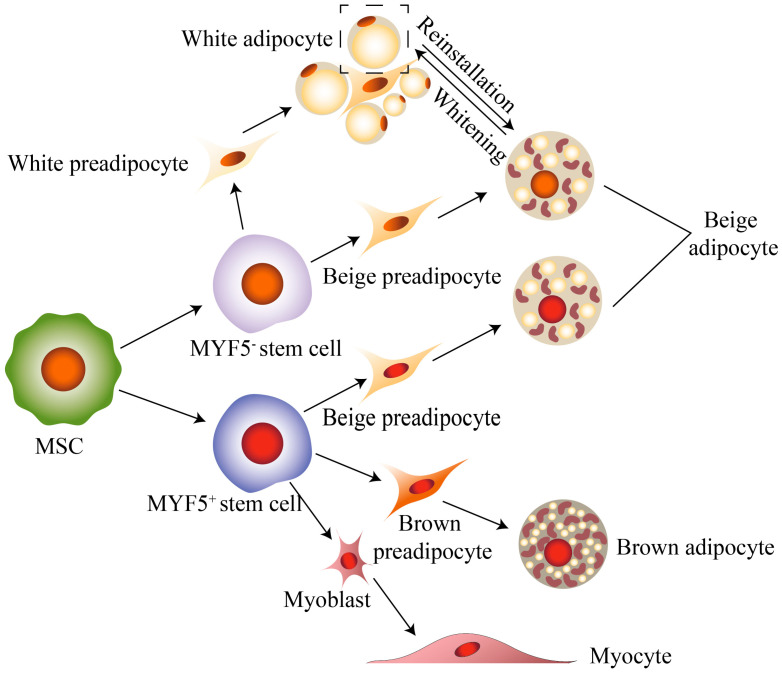
Brown and beige adipocytes’ growth and development. Mesenchymal stem cells (MSCs) differentiate into two types of precursor cells: MYF5^+^ stem cells and MYF5^−^ stem cells. MYF5^+^ stem cells can differentiate into brown precursor and myoblast; MYF5^−^ stem cells can differentiate into white precursor. Interestingly, when exposed to cold stimulation, beige adipocytes produce both MYF5^+^ and MYF5^−^. Beige adipocytes can be transdifferentiated from mature white adipocytes.

Thermogenic adipocytes include brown and beige adipocytes, and they are characterized by their multilocular lipid droplet (LD) morphology and high expression of the mitochondrial protein UCP1. Despite these similarities, brown and beige adipocytes are recognized as two distinct cell types because of differences in their developmental origin, regulation, and function [9] (Figure 1). At an earlier developmental stage, brown adipocytes and myoblast express the myogenic factor 5 (MYF5) gene, which is absent in white adipocytes, suggesting that brown adipocytes may originate from MYF5-expressing precursor cells [30]. As previously mentioned, white adipocytes are induced to produce beige adipocytes under cold stimulation or in response to β-adrenoceptor agonists. However, it is important to note that not all white adipocytes have the ability to differentiate into beige adipocytes. In other words, the precursor cells capable of generating beige adipocytes are different from both brown adipocytes and white adipocytes [29]. It is widely accepted that beige adipocytes are produced from de novo-differentiated adipocytes upon initial stimulation. However, upon subsequent stimulation, they are transdifferentiated from mature white adipocytes.

### 3.1. Brown Adipocytes

According to pulse-chase lineage tracing studies, the fate of brown adipocytes appears to be determined during mid-gestation [31]. Bipotent FOXC^1+^ PAX^3+^ MYF^5+^ paraxial mesoderm (PM)/dermal myotome progenitor cells serve as precursors for brown adipocytes and skeletal muscle cells during embryonic development [32]. In mice, brown adipocytes develop during prenatal development, indicating that their thermogenic function is fully active at birth [33]. From a developmental perspective, embryonic brown adipocytes in mice express somite markers, such as paired-box protein 3 (PAX3), PAX7, mesenchyme homeobox 1 (MEOX1), engrailed 1 (EN1), and MYF5 [30,34,35]. However, it should be noted that all of the aforementioned studies were conducted on mice, limiting our understanding of human BAT development.

Recently, a study showed human pluripotent stem cells (hPSCs) are subject to directed differentiation through a PM progenitor state with the expression of *MYF5* and *PAX3* that generates functional human brown adipocytes at high efficiency [32]. It is evident that this process aligns with the developmental process of brown adipocytes in mice albeit with distinct mechanisms of action. A recent study generated Solute Carrier Family 6 Member 4 (SLC6A4), which encodes serotonin transporter (SERT), can prevent serotonin-mediated suppression of human BAT function. However, SLC6A4 is highly expressed in humans but not in mice [36]. In summary, pluripotent stem cells can be subject to directed differentiation to generate PM progenitors that are MYF5-positive (MYF5^+^) and can continue to undergo directed differentiation to produce precursors for brown adipocytes or skeletal muscle cells.

### 3.2. Beige Adipocytes

Unlike brown adipocytes, beige adipocytes develop postnatally [33]. For example, the mitochondrial *UCP1* gene is not expressed in retroperitoneal fat in newborn mice, but its expression is significant by 10 days of age [37]. By the introduction to the foregoing, brown adipocytes and skeletal muscle cells come from MYF5^+^ precursor cells; then do white adipocytes and beige adipocytes come from MYF5-negative (MYF5^−^) precursor cells? A study demonstrated that white adipocytes and beige adipocytes, found in WAT, originated from both MYF5^+^ and MYF5^−^ precursor cells, and the proportion of cells originating from both cell lines varied in adipose tissue from different parts of the body [38]. Therefore, it is not possible to distinguish beige adipocytes from brown adipocytes based solely on the expression of *MYF5*.

Another intriguing finding is that the application of two gene fate mapping techniques indicates that certain beige preadipocytes may exhibit similarities to smooth muscle cells (SMCs), and mature SMCs can give rise to adipocytes with a thermogenic gene expression signature [39]. Thus, SMCs may be one of the precursor cells of beige adipocytes. The emergence of new brown adipocytes in WAT under specific physiological conditions or drug stimulation is referred to as browning. There are currently two predominant views regarding browning: from de novo-differentiated adipocytes [40] and from mature white adipocytes [41].

#### 3.2.1. From De Novo-Differentiated Adipocytes

In 2013, researchers introduced a doxycycline-inducible, mature adipocyte-specific tracing system in a model known as the Adipo-Chaser mouse [40]. After induction with doxycycline, adipocytes were colored blue, whereas newborn adipocytes failed to be colored blue upon stopping doxycycline feeding [40]. In these mice, they found that most of the beige adipocytes that appeared in subcutaneous white adipose tissue under cold and β3-adrenoceptor agonist stimulation were unable to stain blue, suggesting that they were neodifferentiated rather than transdifferentiated from white adipocytes [40].

#### 3.2.2. Directly from Existing Mature White Adipocytes

In 2015, researchers crossed transgenic mice with tamoxifen-sensitive Cre recombinase under the control of the adiponectin promoter (Adipoq-CreERT2) with the Rosa26-loxP-stop-loxP-tdTomato or Rosa26-loxP-mT-loxP-mG reporter strains in interscapular brown adipose tissue (iBAT) and inguinal white adipose tissue (ingWAT) [41]. The study revealed that the beige adipocytes found in the ingWAT were transdifferentiated from mature white adipocytes [41], and it has been observed that the number of white adipocytes in WAT remains unchanged during browning, indicating that beige adipocyte production does not involve cell division or proliferation. Additionally, no significant changes in DNA levels were observed [42]. These findings suggest that beige adipocyte production is not associated with adipocyte proliferation.

Although these observations may seem contradictory, a study has shown that both direct conversion and de novo biogenesis occur in mice. In contrast to previous studies that employed one single cold treatment, the current study employed two cold treatments, both revealing that beige adipocytes originated from different sources [43]. Specifically speaking, beige adipocytes primarily originate from progenitors through de novo beige adipogenesis upon initial exposure to cold temperatures, and then they interconvert between dormant beige and active beige phenotypes through browning upon subsequent changes in environmental temperature. Furthermore, it is important to note that the majority of experiments were conducted on subcutaneous WAT located in the groin of mice, but it is possible that the production process of beige adipocytes in other areas of subcutaneous and visceral fat may not be entirely consistent. Further in-depth research is required.

## 4. Mechanisms of Non-Shivering Thermogenesis in Beige Adipocytes

### 4.1. Classical UCP1-Dependent Thermogenesis

As is well known, electrons transmit free energy released through the respiratory chain for ATP synthesis in the oxidative phosphorylation process of eukaryotic mitochondria. Unlike this, in the mitochondria of brown adipose tissue, UCP1 can decouple the two processes of electrons transfer and ATP synthesis. However, UCP1 has no constitutive activity [44]. The direct binding of long-chain fatty acids (LFAs) and purine nucleotides to UCP1 can make it have activity and play a role. There exists a competitive inhibition between them, where the binding of di- and triphosphate forms of purine nucleotides (ADP, ATP, GDP, GTP) to UCP1 can inhibit the uncoupling of oxidative phosphorylation in the absence of FFAs [45]. Recently, a novel finding indicated that purinergic/inosine signaling might serve as an approach to increase adipose tissue energy expenditure [46]. The fatty acids used for UCP1 uncoupling are primarily derived from WAT lipolysis rather than thermogenic adipocytes’ lipolysis [47]. Abundant evidence has proved the fact that FFAs serve as both a transport substrate for the solute carrier UCP1 and as a proton shuttle via its terminal carboxyl group [48]. It is also worth mentioning that fatty acid synthesis and oxidation are both stimulated and tightly regulated by the activation of β-adrenergic receptors (β-ARs) [49].

**Figure 2:**
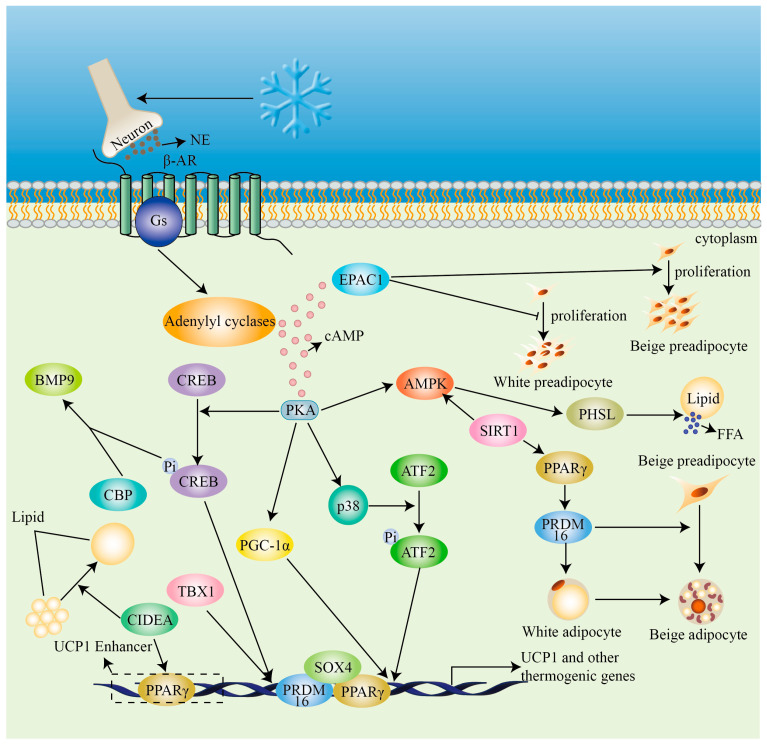
Molecular regulation of heat production in beige adipocytes. In response to cold stimulation, neurons exocytose catecholamines (dark brown circles), which bind to β-adrenoreceptors (β-AR) coupled to the Gs alpha subunit, activating adenylyl cyclases to increase cAMP (pink circles), thereby activating protein kinase A (PKA) and exchange proteins directly activated by cAMP 1 (EPAC1). EPAC1 promotes thermogenic adipocyte proliferation while inhibiting white preadipocyte proliferation. PKA promotes the phosphorylation of a number of genes, resulting in the promotion of transcription of *UCP1* and other thermogenic genes. BMP9 is a protein that activates thermogenesis, or heat production, by modulating brown and beige adipocyte activation. By PKA and AMP-activated protein kinase (AMPK), the phosphorylation of hormone-sensitive lipase (pHSL) can promote lipolysis, which produces free fatty acids (FFAs). SIRT1 can induce the browning of WAT via deacetylation of PPARγ. CIDEA is located in lipids and regulates lipid fusion and enhances PPARγ binding to the Ucp1 enhancer, thereby promoting *UCP1* transcription. SOX4 forms a complex with PRDM16 and PPARγ, which promotes their binding and positively regulates the transcription of the gene UCP1.

Cold exposure stimulates the secretion of the catecholamine norepinephrine (NE) from the sympathetic nervous system (SNS) nerve terminals that are innervated within BAT [50]. β-ARs, coupled to Gs alpha subunits, activate membrane-associated adenylyl cyclases, thereby lead to a rise in cAMP [51]. Increased cAMP activates cAMP-dependent protein kinase A (PKA) phosphorylates cyclic-AMP response binding protein (CREB) and activates p38 mitogen-activated protein kinase (MAPK) for phosphorylating-activating transcription factor 2 (ATF2) [52]. The phosphorylation of CREB and ATF2 can facilitate the transcription of thermogenic genes by directly binding to the cAMP-responsive elements in the promoter region upon cold exposure [53]. The activation of PKA also triggers the activation of AMPK signaling pathways, promoting WAT lipolysis by hormone-sensitive lipase (pHSL) phosphorylation and results in the direct transcriptional activation of *Ppargc1a* (encoding peroxisome proliferator-activated receptor-γ (PPARγ) co-activator-1, PGC1α), and *UCP1* [54] (Figure 2).

### 4.2. Novel UCP1-Independent Mechanisms

**Figure 3:**
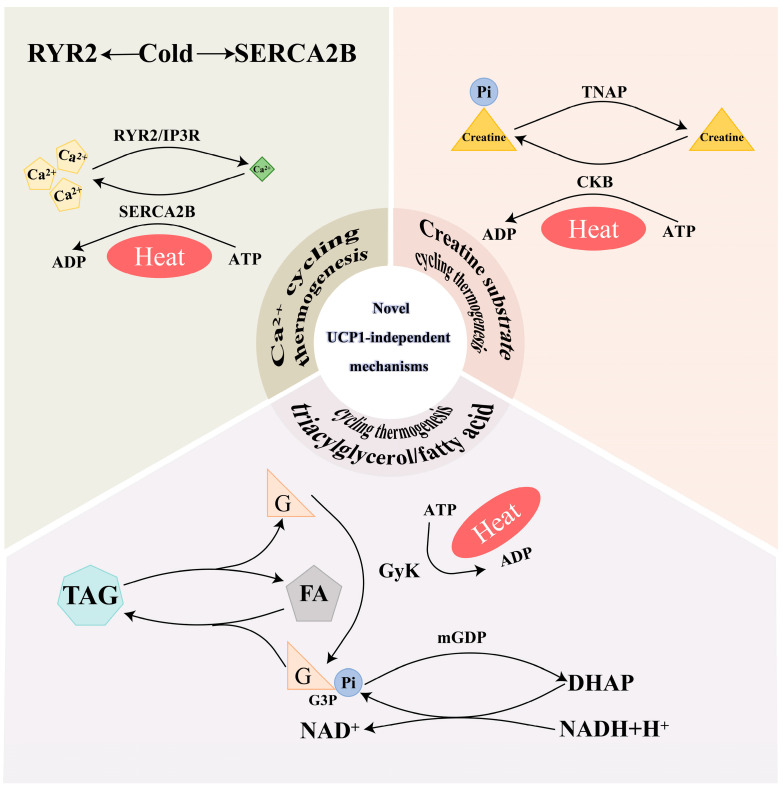
The three novel UCP1-independent mechanisms: Ca^2+^ cycling thermogenesis, creatine substrate cycling thermogenesis, and triacylglycerol/fatty acid cycling thermogenesis. ATP, adenosine triphosphate; ADP, adenosine diphosphate; Pi, phosphate; RYR2, ryanodine receptor 2; IP3R, inositol trisphosphate receptor; SERCA2B, sarco/endoplasmic reticulum Ca ^2+^-ATPase 2B; TNAP, tissue-non-specific alkaline phosphatase; CKB, creatine kinase B; TAG, triacylglycerol; FAs, fatty acids; G, glycerol; GyK, glycerol kinase; G3P, glycerol 3-phosphate; mGDP, mitochondrial G3P dehydrogenase; DHAP, dihydroxyacetone-3-phosphate; NAD+, nicotinamide adenine dinucleotide; NADH, nicotinamide adenine dinucleotide.

As a result of the above, there is no doubt that UCP1 plays an important role in non-shivering thermogenesis (NST). UCP1-deficient mice induce alternative thermogenic mechanisms that emerge through enhanced ion and substrate cycling associated with brown adipocytes in white fat depots by gradual cold adaptation [55], and the chronic administration of β3-adrenergic agonists to *UCP1* KO mice increases oxygen consumption in inguinal and epididymal WAT [49]. Despite their extreme sensitivity to acute cold exposure, *UCP1* KO mice can be gradually adapted to temperatures as low as 4 °C [56]. In addition, while pigs inherently lack *UCP1*, cold-tolerant breeds, such as Min and Tibetan pigs from China, still exist [11]. The above evidence suggests the presence of UCP1-independent mechanisms from the perspective of animal models. Ca^2+^ cycling and creatine-substrate cycling have been mentioned more often so far (Figure 3).

#### 4.2.1. Ca^2+^ Cycling Thermogenesis

Calcium ions are primarily present in a free form within cells and are unevenly distributed throughout various organelles and the cytoplasmic matrix. The concentration of calcium ions in the endoplasmic reticulum under physiological conditions ranges from 100 to 800 μmol/L, while the concentration of free calcium ions in the cell matrix is approximately 100 nmol/L. In contrast, the extracellular calcium ion concentration may increase up to 2 mmol/L. This large concentration disparity provides a basis for calcium ion signal transduction [57]. Sarco/endoplasmic reticulum Ca^2+^-ATPase (SERCA) functions as a crucial regulator. With the participation of SERCA, Ca^2+^ in the cytoplasmic matrix is actively transported to the endoplasmic reticulum (ER) through the reverse concentration gradient. This part of the energy is provided by ATP hydrolysis. In the presence of Ca^2+^ release channels, ryanodine receptors (RYRs), and inositol trisphosphate receptor (IP3R), Ca^2+^ is transported from the ER back into the cytoplasmic matrix. The above process constitutes Ca^2+^ cycling [58]. SERCA is divided into four subtypes: SERCA1, SERCA2a, SERCA2b, and SERCA3. Sarcolipin (SLN) is a micropeptide that binds to SERCA1 in skeletal muscle, uncoupling ATP hydrolysis from Ca^2+^ pumping into the sarcoplasmic reticulum and promoting NST [59]. SERCA2b facilitates the release of substance Ca^2+^ from the ER into the cytoplasmic matrix by RYR2 [60]. Ca^2+^ cycling thermogenesis occurs mainly in skeletal muscle and beige adipocytes.

Several metabolic pathways, including tricarboxylic acid (TCA) metabolism, branched-chain amino acid (BCAA) oxidation, and glycolysis, were uniquely up-regulated in PRDM16 Tg x UCP1−/− mice compared to the PRDM16 Tg mice and UCP1−/− mice. It can be inferred that there is a UCP1-independent metabolic mechanism in beige adipocytes that contributes to systemic energy and glucose homeostasis. Notably, the mRNA expression of several cardiac muscle-related genes involved in Ca^2+^ cycling was uniquely up-regulated, but unlike skeletal muscle, the expression of SERCA2b in the SERCA isoform in beige fat is significantly higher than that of SERCA1. Under the stimulation of cold exposure, the α1-AR is activated, which triggers intracellular Ca^2+^ influx with the participation of SERCA2b; at the same time, the β3-AR signaling is also activated, which triggers Ca^2+^ release from the endoplasmic reticulum by promoting RYR2 activity. Notably, this thermogenic mechanism is necessary for beige adipocyte thermogenesis, but it is dispensable in brown adipocytes [60]. This may be the reason why beige adipocytes possess higher ATP synthesis capacity compared to brown adipocytes [61]. It can be deduced that maintaining a large concentration difference between Ca^2+^ in the cytoplasmic matrix and the ER can promote ATP hydrolysis, thus narrowing the concentration difference. But humans RYR2 gene mutations cause arrhythmogenic right ventricular cardiomyopathy type 2 and lethal arrhythmia due to catecholaminergic polymorphic ventricular tachycardia [25]. Therefore, the systematic activation of Ca^2+^ cycling is detrimental, and the selective activation of Ca^2+^ cycling in beige fat represents a promising research direction.

#### 4.2.2. Creatine Substrate Cycling Thermogenesis

In 1976, it was proposed that the energy metabolism of adipose tissue is closely related to the presence of creatine [62]. According to the study, the phosphocreatine(PCr)/creatine(Cr) ratio in beige adipocytes is reduced significantly in cold-exposed mice compared to their thermoneutral counterparts, but there is no difference in BAT and skeletal muscle [63]. Hence, the futile cycle of PCr and Cr is proposed under ADP-limiting conditions. The cycle involves two steps. First, tissue-non-specific alkaline phosphatase (TNAP) hydrolyzes PCr and generates Cr and inorganic phosphate (Pi). Second, creatine kinase B (CKB) catalyzes the transfer of phosphoryl from mitochondrial ATP to creatine to simultaneously generate PCr and release ADP [64]. Remarkably, TNAP is localized to the mitochondria of thermogenic adipocytes, and CKB constitutes the major creatine kinase activity in brown adipocytes [64,65]. Furthermore, glycine amidino transferase (GATM) is the first rate-limiting enzyme of creatine biosynthesis, and adipo-Gatm KO mice are prone to obesity in response to high-calorie feeding [66]. These studies suggest creatine transporter (CrT, also known as Slc6a8) [67] and zinc-finger protein Zfp423 [68] affect thermogenic respiration. Nevertheless, there have been voices opposing futile creatine cycling (FCC), because there is currently no convincing evidence to support that a futile creatine cycle is related to NST [69].

#### 4.2.3. Triacylglycerol/Fatty Acid Cycling Thermogenesis

The triacylglycerol/fatty acid cycling (TAG/FA cycling), a substrate cycle commonly used for thermogenesis, is formed by simultaneous lipolysis and re-esterification. The sequential hydrolysis of TAG is catalyzed to generate glycerol and fatty acids (FAs) by three different enzymes, including adipose triglyceride lipase (ATGL), hormone-sensitive lipase (HSL), and monoacylglycerol lipase (MGL) [70]. Next, glycerol kinase (GyK) phosphorylates glycerol to glycerol 3-phosphate (G3P), which is a primary backbone for TAG [71]. These FA and G3P can also be re-esterified back to TAG, resulting in futile TAG/FA cycling in adipocytes. Notably, the G3P is reoxidized to dihydroxyacetone-3-phosphate (DHAP) by the mitochondrial G3P dehydrogenase (mGPD), which is highly expressed in BAT [72]. According to the study, there is modestly reduced energy expenditure in transgenic mice lacking the *mGPD* gene (mGPD−/−) [73]. This is because DHAP serves as an acceptor of NADH generated in the cytoplasm, resulting in the transfer of NADH to complex III rather than complex I of the mitochondrial respiratory chain, resulting in the generation of two ATPs per oxygen atom. Then, *mGPD−/−* will affect the synthesis of ATP, and the *mGPD−/−* mice have shown reduced energy expenditure.

Of interest, the *UCP1* and *mGPD* double KO mice exhibited higher energy expenditure than the control group, which was accompanied by increased beige fat biogenesis and respiration in inguinal WAT (iWAT) [74]. Accordingly, the *UCP1* and *mGDP* double KO may facilitate the development of alternative thermogenic pathways to compensate for the loss of the aforementioned thermogenic pathways. However, the specific nature of thermogenic pathways, whether it be Ca^2+^ cycling thermogenesis, creatine substrate cycling thermogenesis, or other compensatory mechanisms, remains unclear. A recent study provides direct evidence of the presence of the TAG/FA cycling, emphasizing the important role of the TAG cycling in monitoring and maintaining the composition of FA stored in adipocytes [75].

## 5. Important Genes of Thermogenesis in Beige Adipocytes

### 5.1. UCP1

Uncoupling proteins (UCPs), which facilitate mitochondrial proton H^+^ transport and participate in oxidative phosphorylation uncoupling, are a type of carrier protein that exhibit tissue-specific expression. In mammals, UCPs exhibit tissue-specific expression and have six members in mammals: UCP1, UCP2, UCP3, UCP4, UCP5, and UCP6 [76,77,78,79,80,81]. *UCP1* is an important gene that is located in the mitochondrial inner membrane and releases the electrochemical potential energy generated by the high concentration of protons on the cytoplasmic side as thermal energy, contributing to NST [82]. Similarly, UCP1 is expressed in beige adipocytes and can perform the same function as it does in brown adipocytes. As previously stated, UCP1 is inactive by itself and requires activation to perform its function. Intriguingly, pigs lack functional UCP1 due to the absence of exons 3–5 but exhibit beige adipocytes by FDG-PET/CT during cold exposure [11]. This indicates the presence of UCP1-independent mechanisms in beige fat.

### 5.2. DIO2

The role of the type 2 iodothyronine deiodinase (DIO2) in the activating of thyroid hormones impacts the expression of UCP1 [83]. The specific process by which DIO2 promotes thermogenesis is as follows: DIO2 can enhance the intracellular activation of thyroxine (T4) to 3,3′,5-triiodothyronine (T3) [84]. Thyroid hormone receptor α (TRα) mediates the T3-induced up-regulation of UCP1 protein expression in brown adipocytes, thereby promoting thermogenesis [85]. A study has shown that cold-exposed mice with targeted disruption of the *DIO2* gene become hypothermic [84].

### 5.3. CIDEA

Cell death-inducing DNA fragmentation factor, alpha subunit-like effector A (CIDEA), is a lipid-droplet-associated protein and shows a rhythmic expression pattern [86]. In 2017, it was demonstrated that *CIDEA* was identified as a marker gene for beige adipocytes based on both mRNA and protein levels [87]. CIDEA is expressed endogenously in white adipocytes of humans but not in mice [87]. But it is important to note that there are two views on the role of CIDEA: one is that CIDEA inhibits UCP1 activity, while the other one is the exact opposite. CIDEA-null mice exhibit a lean phenotype and are resistant to diet-induced obesity and diabetes [88]. And the study concluded that CIDEA is a mitochondrial protein and forms a complex with UCP1 that can attenuate the uncoupling activity [89]. Additionally, CIDEA is subsequently located in LDs and regulates LD fusion [90]. Recently, it is revealed that calsyntenin-3β (CLSTN3β) localizes to ER–LD contact sites, where it promotes lipid droplet multilocularity by blocking CIDEA-mediated LD fusion [91]. All of the studies mentioned above demonstrate that the CIDEA substance inhibits beige adipocytosis and thermogenesis in mice. However, in human white adipocytes, CIDEA localizes to both the cytoplasm and the nucleus [92]. And the region of the UCP1 promoter/enhancer has two binding sites: the PPARγ-binding site and Liver X Receptorα (LXRα)-binding site [93]. It has been shown that CIDEA specifically inhibits UCP1 enhancer activity by LXRα and enhances PPARγ binding to the UCP1 enhancer, thereby promoting *UCP1* transcription [93]. However, it should be noted that the above studies are limited to in vitro experimentation and require further validation in an in vivo physiological environment.

### 5.4. SIRT1

Resveratrol is a natural polyphenolic compound that can activate silent mating type information regulation 2 homolog 1 (SIRT1), which controls the deacetylation of proteins [94]. SIRT1 has an NAD^+^-dependent deacylation of acyl-lysine residues [95]. SIRT1 promotes increased mitochondrial biogenesis and UCP1 expression and the browning of white adipocytes through the deacetylation of PGC-1α [96,97]. Moreover, SIRT1 also can induce the browning of white adipocytes via the deacetylation of PPARγ or by binding to nuclear receptor corepressor 1 (NCOR1) [89]. Corylin, a flavonoid, can enhance SIRT1 activity by forming a strong hydrogen bond with SIRT1. And the present study demonstrated that corylin induced the browning of WAT and lipolysis through an SIRT1-dependent pathway [98].

### 5.5. FGF21

Fibroblast growth factor 21 (FGF21) serves as a stress-responsive hormone that interacting with β-klotho (KLB) and fibroblast growth factor tyrosine kinase receptor (FGFR). BAT relies on the FGFR1/KLB complex to respond to FGF21. This induces glucose uptake and thermogenesis through the induction of UCP1 in response to its autocrine and paracrine production [99]. Elevated expression of *FGF21* was detected at both the mRNA and protein levels during beige lipogenesis, and the protein was detected in the culture medium. This study provided evidence that FGF21, originating from beige adipocytes, acts as a paracrine/endocrine factor in humans [100]. And FGF21 is mainly produced in adipose tissue and liver. It is worth noting that the autocrine signaling of adipose-derived FGF21 promotes the thermogenic gene expression in WAT, while liver-derived FGF21 is not necessary for the beiging of WAT [101]. Overall, FGF21 can enhance the rate of adipose tissue browning and energy expenditure, and its beneficial metabolic effects can also occur in UCP1-independent mechanisms [102].

### 5.6. BMP9

Bone morphogenetic protein 9 (BMP9), also known as growth differentiation factor 2 (GDF2), is a hepatokine. Transcriptional activation induced by CREB and CREB binding protein (CBP) on the *BMP9* promoter regulates BMP9 mRNA expression in the liver. Moreover, some of the other genes in this family, such as *BMP4*, *BMP7*, and *BMP8B*, have been shown to be activators of thermogenesis through regulating brown or beige adipocyte activation [103]. Studies have identified BMP9 as the most potent osteogenic factor for inducing osteogenic differentiation. Another study suggests that BMP9-induced adipogenic capability may follow the following order: immortalized mouse adipose-derived mesenchymal stem cells (iMAD) ≥ immortalized mouse bone marrow stromal stem cells (imBMSC) > immortalized mouse embryonic fibroblasts (iMEF) ≥ immortalized mouse calvarial mesenchymal progenitors (iCAL) cells in vitro [104]. However, the mechanism of action need to be further revealed.

### 5.7. EPAC1

Exchange proteins directly activated by cAMP (EPACs) act as downstream effectors of cAMP [105]. Recent research has demonstrated that EPAC1 promotes the proliferation of thermogenic adipocytes while inhibiting the proliferation of white preadipocytes. Moreover, EPAC1 activation increased ERK1/2 (an important initiator and regulator of mitosis) phosphorylation and the expression of C/EBPβ only in brown preadipocytes. Thus, EPAC1 is considered a significant regulator of brown/beige adipocyte proliferation and differentiation [106].

## 6. Main Transcription Factors of Thermogenesis in Beige Adipocytes

### 6.1. PGC-1α

PPARγ coactivator-1α(PGC-1α) is the initial transcriptional coactivator of nuclear receptors that is primarily located in mitochondria-rich tissues such as adipose, liver, skeletal muscle and heart, to be discovered in adaptive thermogenesis and is a key regulator of lipid and energy metabolism [107,108]. PPARγ is highly expressed in adipose tissue and is essential for adipose differentiation and fat metabolism [109]. PGC-1α exerts its adaptive thermogenesis mainly through the nuclear receptor PPARγ under conditions of cold exposure, exercise, or fasting [94]. Moreover, PGC-1α is essential for the activation of beige adipocytes induced by cold exposure or β3-adrenergic agonists [110,111]. For instance, in a study involving mice treated with rosiglitazone (PPARγ agonist) in mice, there was a significant increase in the expression of UCP1 and PGC-1α in WAT [112]. And the overexpression of PGC-1α in white adipocytes can induce the expression of UCP1 and key mitochondrial enzymes of the respiratory chain [107].

### 6.2. PRDM16

PR domain-containing 16 (PRDM16) is a transcription factor that regulates the browning of white adipocytes by increasing mitochondrial biogenesis and activating several transcription factors [113]. Furthermore, PRDM16 interacts with zinc finger protein 516 (ZFP516) and PPARγ to activate the UCP1 promoter, which is involved in the activation of thermogenic genes in brown and beige adipocytes, promoting adipose tissue thermogenesis [114,115]. A recent study has shown that the expression of PRDM16 protein decreased with age, while its mRNA expression remained unchanged [116]. Significantly, the study found that the ubiquitin E3 ligase CUL2–APPBP2 determines PRDM16 protein stability by catalyzing PRDM16 polyubiquitination in beige fat [116]. Accordingly, the post-translational control of PRDM16 has an impact on the biogenesis and function of beige fat.

### 6.3. TBX1

The transcription factor T-box 1 (TBX1) whose relative mRNA expression was significantly higher in beige adipocytes compared to white and brown adipocytes, as determined by qPCR, has been utilized as a marker for beige adipose tissue [29]. The expression of TBX1 in adipose tissue is necessary for maintaining body temperature during cold exposure, but the transgenic overexpression of TBX1 specifically in adipose tissue is not sufficient to induce the browning of WAT [117]. Another member of this family, T-box 15 (TBX15), regulates adipocyte browning by directly binding to a key region in the *PRDM16* promoter [118]. Although sharing a conserved DNA binding T-box domain with *TBX15*, *TBX1* is neither a strong transcriptional repressor nor activator. Instead, it exerts effects on transcription through off-DNA protein–protein interactions and promotes H3K4me1 deposition [117]. Nevertheless, the mechanism of action of TBX1 is currently unknown.

### 6.4. SOX4

Sex-determining region Y-related high-mobility-group box transcription factor 4 (SOX4) is thought to be an important transcription factor. The gene was initially thought to be associated with type 2 diabetes (T2D) and obesity [119]. Recent studies revealed that SOX4 forms a complex with PRDM16 and PPARγ, promoting their binding and positively regulating the transcription of the gene *UCP1* [120]. The mechanism of action has been revealed.

## 7. Specific Markers of Beige Adipocytes

### 7.1. CD137

The transmembrane protein CD137, also referred to as Tnfrsf9 and 4–1BB, is a member of the tumor necrosis factor (TNF) receptor superfamily [121]. In microarray screens of immortalized cell lines derived from the iWAT of obesity-resistant Sv129 mice, *CD137* was identified as a marker for beige preadipocytes [29]. Several studies have reported higher expression levels of *CD137* in the beige preadipocytes with in the SVF, but its expression decreases in mature beige adipocytes [122,123]. Besides, most studies on *CD137* have focused on mRNA levels, neglecting protein levels, leading to controversy regarding the suitability of CD137 as a marker for beige adipocytes. In the first place, the high mRNA expression of *CD137* in SVF may be attributed to inflammatory stimuli [124], and it is difficult to identify changes in transcript levels solely due to beige adipogenesis. What is more, although *CD137* mRNA expression was increased when cultured cells from the SVF were differentiated into beige adipocytes, interestingly, the levels of CD137 protein were below detection levels [121]. In the third place, in *CD137* knockout mice, the number of beige preadipocytes was increased and cold resistance was enhanced, which was unexpected [121]. Nevertheless, no studies have used the tissue-specific knockout of *CD137* to investigate its specific impact on the formation and function of beige adipocytes.

### 7.2. TMEM26

Transmembrane protein 26 (TMEM26) is specifically detected in beige adipocytes, making it a marker for beige adipocytes [29]. However, it is worth noting that the mRNA expression of *TMEM26* was lower in beige adipocytes when compared to SVF [123]. The mRNA expression of *TMEM26* was not increased by either adrenergic or cold stimulation [123]. Despite this, *TMEM26* remains one of the beige adipocyte-specific genes [125,126,127]. To date, it remains unclear that what function *TMEM26* has in beige adipocytes.

### 7.3. CITED1

Cbp/p300 interacting transactivator with Glu/Asp rich carboxy-terminal domain 1 (CITED1) encodes a 27 kDa nuclear protein and coregulates transcriptional nuclear proteins via their transactivator domains [128]. CITED1 was originally described as a transcriptional coregulator of estrogen receptor α (ER α) in non-neuronal cells [129]. Recently, a study has indicated that CITED1 contributes to leptin’s anorectic effects by acting as a co-factor that converges estradiol and leptin signaling through direct Cited1-estrogen receptor α-signal transducer and activator of transcription 3 interactions in terms of animal behavior, and the way conserves between sexes [130]. In 2012, *CITED1* was identified as a new beige adipocytes marker by qRT-PCR in rosiglitazone-treated adipocytes [131]. Currently, *CITED1* is widely used as a marker gene for beige adipocytes [132,133,134]. However, there are currently no reports on the specific role of CITED1 in beige adipocyte differentiation.

## 8. Conclusions and Perspective

In recent years, thermogenic adipose tissue has attracted widespread attention for several reasons, primarily due to the increasing prevalence of obesity and the higher risk of its associated metabolic diseases. Notably, thermogenic adipose tissue exhibits the potential to inhibit weight gain. Additionally, studies have revealed that obese individuals posses significantly lower levels of thermogenic adipose tissue compared to their non-obese counterparts. Animals with high thermogenic adipose tissue content show excellent cold tolerance under low-temperature environment, a trait that has attracted the interests from the livestock industry due to the potential for cold stress response in certain animals, such as piglets, when raising livestock and poultry in a low-temperature environment. However, the amount of BAT in thermogenic adipose tissue is determined before birth and decreases with age. Therefore, beige adipose tissue, which is non-dermal musculature-derived thermogenic adipose tissue that can be recruited after birth, has emerged as a promising target for further study.

However, studying beige adipocytes presents a challenge due to their similarity in morphological to white adipocytes, even under ideal conditions. The advancement of single-cell analysis techniques combined with cellular lineage determination has revealed that adipocytes have multiple subtypes. Specifically, beige adipocytes are produced under different triggering conditions. For instance, beige adipocytes produced by cold stimulation differ from those produced by β3-AR agonist induction, as cold-induced formation of beige adipocytes relied on β1-AR agonist rather than β3-AR agonist [135]. Secondly, beige adipocytes need to be induced by external stimuli such as cold stimulation, exercise, and diet. However, the activation of beige adipose tissue thermogenesis by cold stimulation or β-adrenergic signaling may lead to potential cardiovascular disease [136]. Thirdly, current tests cannot distinguish beige adipose tissue from tumors. And unlike metabolic diseases, the effect of thermogenic adipose tissue on cancer is not unilaterally beneficial or detrimental but rather a double-edged sword. The activation of beige adipose tissue in the vicinity of a tumor can promote the proliferation and invasion of cancer cells [137].

In general, beige adipose tissue has great potential for research as an acquired thermogenic adipose tissue. Compared to BAT, beige adipose tissue has more diverse thermogenic mechanisms and cellular sources, also exhibiting greater reponsiveness to external environment to adapt to various environmental changes. In recent years, most molecular genetic and epigenetic studies of beige adipose tissue have focused on the regulation of UCP1 expression. However, recent studies have shown that thermogenesis in brown adipocytes is regulated by two parallel pathways, UCP1 and creatine kinase B (CKB), indicating that classical UCP1-dependent thermogenesis is not the sole mechanism [138]. Therefore, further research is imperative to expand our understanding of thermogenic adipose tissue, and the application of more advanced technologies in the future may provide deeper insights into its underlying the mechanisms, thus paving a novel path for maintain human health.

## Data Availability

No new data were created or analyzed in this study. Data sharing is not applicable to this article.
